# Supplementary material for: Complete Sequences of the Velvet Worm Slime Proteins Reveal that Slime Formation is Enabled by Disulfide Bonds and Intrinsically Disordered Regions
Source: Adv Sci (Weinh). 2022 May 18;9(18):2201444. doi: 10.1002/advs.202201444 (PMC9218773; doi:10.1002/advs.202201444)
Supplement: Supplementary file 1 — Supporting Information [file ADVS-9-2201444-s005.pdf]

ADVANCED SCIENCE
Open Access

Supporting Information

for *Adv. Sci.*, DOI 10.1002/advs.202201444

Complete Sequences of the Velvet Worm Slime Proteins Reveal that Slime Formation is Enabled by Disulfide Bonds and Intrinsically Disordered Regions

*Yang Lu, Bhargy Sharma, Wei Long Soon, Xiangyan Shi, Tianyun Zhao, Yan Ting Lim, Radoslaw M. Sobota, Shawn Hoon, Giovanni Pilloni, Adam Usadi, Konstantin Pervushin and Ali Miserez**

Supporting Information

Complete sequences of the velvet worm slime proteins reveal that slime formation is enabled by disulfide bonds and intrinsically disordered regions

*Yang Lu,[#], Bhargy Sharma,[#], Wei Long Soon, Xiangyan Shi, Tianyun Zhao, Yan Ting Lim, Radoslaw M. Sobota, Shawn Hoon, Giovanni Pilloni, Adam Usadi, Konstantin Pervushin and Ali Miserez.**

Supplementary Tables

Table S1. Single polymorphism as capital letter of ES_P1 and ES_P2 detected in Re-sequencing with designed primers comparing to the sequences obtained from RNAseq and the related changes in amino acid composition.

	Position of RNAseq	RNAseq	Amino acid	Re-seq with primers	Amino acid
ES_P1	73	aGa	Arg	aCa	Thr
	409	cCt	Pro	cTt	Leu
	447	Ctt	Leu	Ttt	Phe
	519	Gta	Val	Ata	Ile
	527	acA	Thr	acT	Thr
	701	ccT	Pro	ccC	Pro
	790	aCt	Thr	aTt	Ile
	893	gtC	Val	gtT	Val
	969	Cgt	Arg	Ggt	Gly
	1517	ggC	Gly	ggA	Gly
	1975	aGa	Arg	aAa	Lys
	2734	aAa	Lys	aGa	Arg
	2795	aaA	Lys	aaC	Asn
	2936	ccA	Pro	ccT	Pro
	2937	Aat	Asn	Cat	His
	2951	ttC	Phe	ttT	Phe
	2952	AAA	Lys	Gaa	Glu
	2959	aTa	Ile	aGa	Arg
	2963	ccA	Pro	ccT	Pro
	2995	gAA	Glu	gTG	Val
	2999	ccA	Pro	ccT	Pro
	3002	atA	Ile	atT	Ile

	3008	ccT	Pro	ccA	Pro
	3011	gaT	Asp	gaA	Glu
	3012	Gat	Asp	Aat	Asn
	3023	gaG	Glu	gaT	Asp
	3024	CCa	Pro	GAa	Glu
	3032	gAC	Asp	gaT	Asp
	3038	gaA	Glu	gaT	Asp
	4028	ggG	Gly	ggA	Gly
	4091	aaC	Asn	aaT	Asn
	4203	Cca	Pro	Gca	Ala
	4784	AAA	Lys	aaG	Lys
	5234	aaT	Asn	aaA	Lys
	5850	Ata	Ile	Gta	Val
	5914	gCg	Ala	gTg	Val
	5923	aCt	Thr	aTt	Ile
**ES_P2**	152	Gat	Asp	Aat	Asn
	1650	cAt	His	cGt	Arg

Table S2. Amino acid analysis of slime comparing sequences ES_P1 and ES_P2.

**Amino acid**	**% in ES slime**	**ES_P1**	**ES_P2**
Aspartate/Asparagine	8.8 ± 0.2	11.3	12.3
Glutamate/Glutamine	11.5 ± 0.0	10.1	8.7
Threonine	3.9 ± 0.2	6.7	4.1
Serine	4.7 ± 0.7	5.2	6.7
Tyrosine	2.6 ± 0.4	3.4	3.9
Phenylalanine	1.9 ± 0.2	3.4	3
Proline	4.3 ± 0.1	17.7	16
Hydroxyproline	0.4 ± 0.0	3.5	NA
Glycine	27.1 ± 0.8	9	9.6
Cysteine	1.9 ± 0.1	0.3	0.2
Alanine	4.8 ± 0.2	1.9	2.6
Valine	4.3 ± 0.5	5.6	5.6
Methionine	1.0 ± 0.2	0.6	0.7
Isoleucine	2.7 ± 0.1	3.7	6
Leucine	4.3 ± 0.1	3.2	5.7
Histidine	3.8 ± 0.3	2.9	1.6
Lysine	8.5 ± 0.2	10.2	9.2
Arginine	3.6 ± 0.2	4.8	3.6

Table S3. Proteins detected in different complex from LC MS/MS.

Position in native gel	Proteins detected
Band_1	ES_P1, ES_P5
Band_2	ES_P1, ES_P5, ES_P6
Band_3	ES_P1, ES_P5
Band_4	ES_P2, ES_P5

Table S4. ^{13}C peak shifts in ppm assigned for amino acid residues in *Eoperipatus sp.* slime based on the average chemical shifts.

<table>
<tr><th>Amino acid</th><th>Cβ</th><th>Cγ</th><th>Cδ</th><th>Cε</th><th>Cζ</th></tr>
<tr><td>Ile</td><td>36.1</td><td>17.4</td><td>14.1</td><td></td><td></td></tr>
<tr><td>Val</td><td>32.2</td><td>21.4</td><td></td><td></td><td></td></tr>
<tr><td>Thr</td><td>72.6</td><td>21.4</td><td></td><td></td><td></td></tr>
<tr><td>Leu</td><td>42.1</td><td></td><td>25.2</td><td></td><td></td></tr>
<tr><td>Lys</td><td>32.2</td><td>25.2</td><td></td><td>42.1</td><td></td></tr>
<tr><td>Glu</td><td></td><td>36.1</td><td>183.8</td><td></td><td></td></tr>
<tr><td>Pro</td><td>32.2</td><td>27.2</td><td>50.8</td><td></td><td></td></tr>
<tr><td>Ser</td><td>63.9</td><td></td><td></td><td></td><td></td></tr>
<tr><td>Asp</td><td>38.2</td><td>180.2</td><td></td><td></td><td></td></tr>
<tr><td>Phe</td><td></td><td>139.2</td><td colspan="3">129.8-132.4</td></tr>
<tr><td>Tyr</td><td></td><td colspan="2">129.8-132.4</td><td>118.2</td><td rowspan="2">159.5</td></tr>
<tr><td>Arg</td><td></td><td></td><td></td><td></td></tr>
</table>

Supplementary Figures

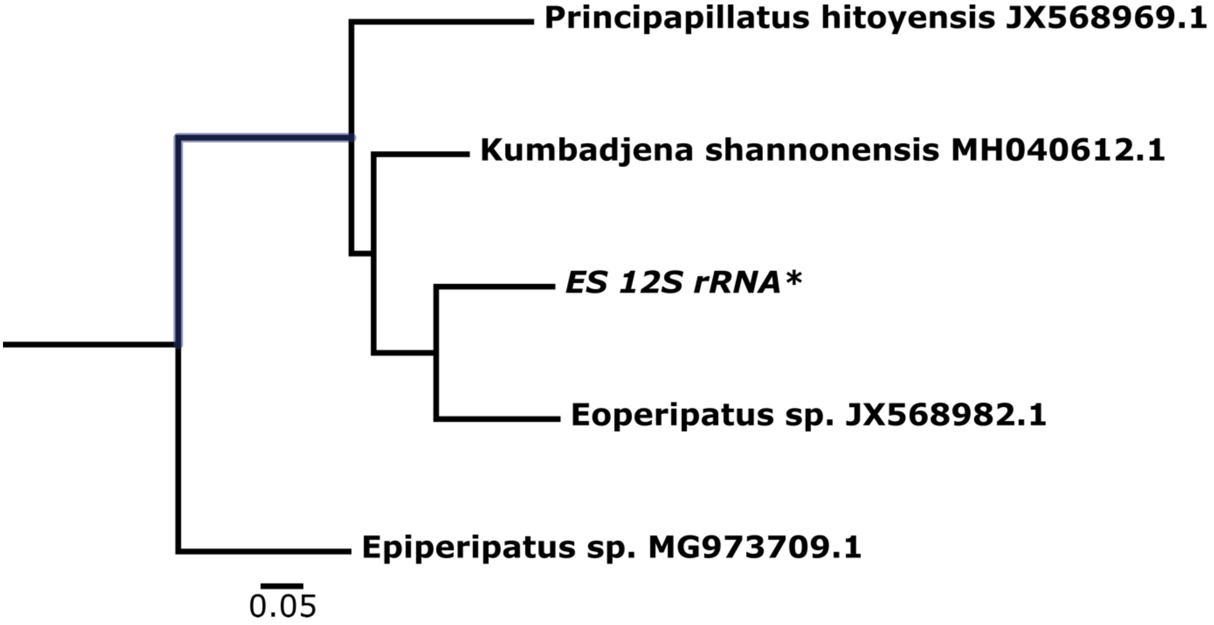

Figure S1. A partial phylogenetic tree indicating the position of ES 12S rRNA in the phylum of Onychophora. Scale bar indicates the phylogenetic distance of 0.05 nucleotide substitutions per site. *Epiperipatus sp*. MG973709.1 is used as root.

```
FUS-LC   MASNDYTQQATQSYGAYPTQPGQGYSQQSSQPYGQQSYSGYSQSTDTSGYGQSSYSSYGQ 60
ES_P1-NT MK-----ILLSVLVLLIVVEC--GNSRKI-RH-------------------RGGSR---R 30
ES_P2-NT ME-----MMYTLFFLLFGIVHGQGDGWVL-QP-------------------DGSYMSYGD 35
         *         :            * .    :                     ..

FUS-LC   SQNTGYGTQSTPQGYGSTGGYGSSQSSQSSYGQQSSYPGYGQQPAPSSTSGSYGSSSQS- 119
ES_P1-NT --------GSGGGSGGSSGGS--SGG----------------------SDGSYGGSDGGS 58
ES_P2-NT --------GSSGGSYGSTGGS--YDG----------------------SGGLYGGSSGGS 63
                  *   . **:**     .                      :.* **.*. .

FUS-LC   -SSYGQPQSGSYSQQPSYGGQQQSYGQQQSYNPPQGYGQQNQYNSSSGGGGGGGGGNYG 178
ES_P1-NT GGSYGDSGGGSGDSTGSNGG-------------------PGDSYSESGGSSGDGGSGGSYG 100
ES_P2-NT ---YGELGSGG-------------------------------LFGGS--GGGGFGPGGSYG 88
            **:  .*.                               :. *  ..*. * **.**

FUS-LC   QDQSSMS-------------------------SGGGSGGGYGNQDQSGGGGSGGYGQQDRG- 214
ES_P1-NT GSDGGPGGSYGG--------------------SGGSGGGGGGGGGGGGSGSDNNPPEGY 141
ES_P2-NT GFDGGLGGSSGGSQGLPGNGWILQPDGSYLKYEGSG--GGGGGGGGGGSGSDGPPGNGW 146
           :.. .                          ***   *. . .******. .   .*
```

Figure S2. Sequence alignment for FUS-LC domain with N-terminal sequence of ES_P1 and ES_P2 proteins.

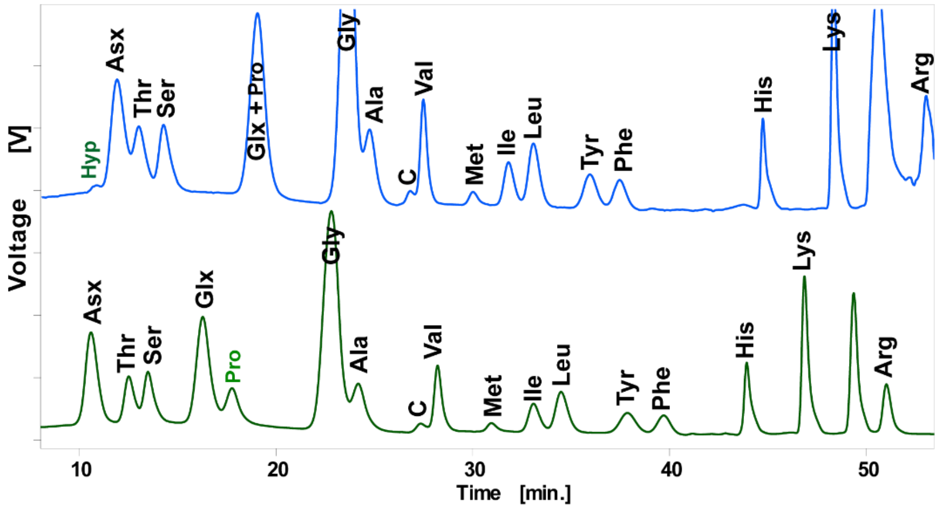

Figure S3. Amino acid chromatogram of the native slime.

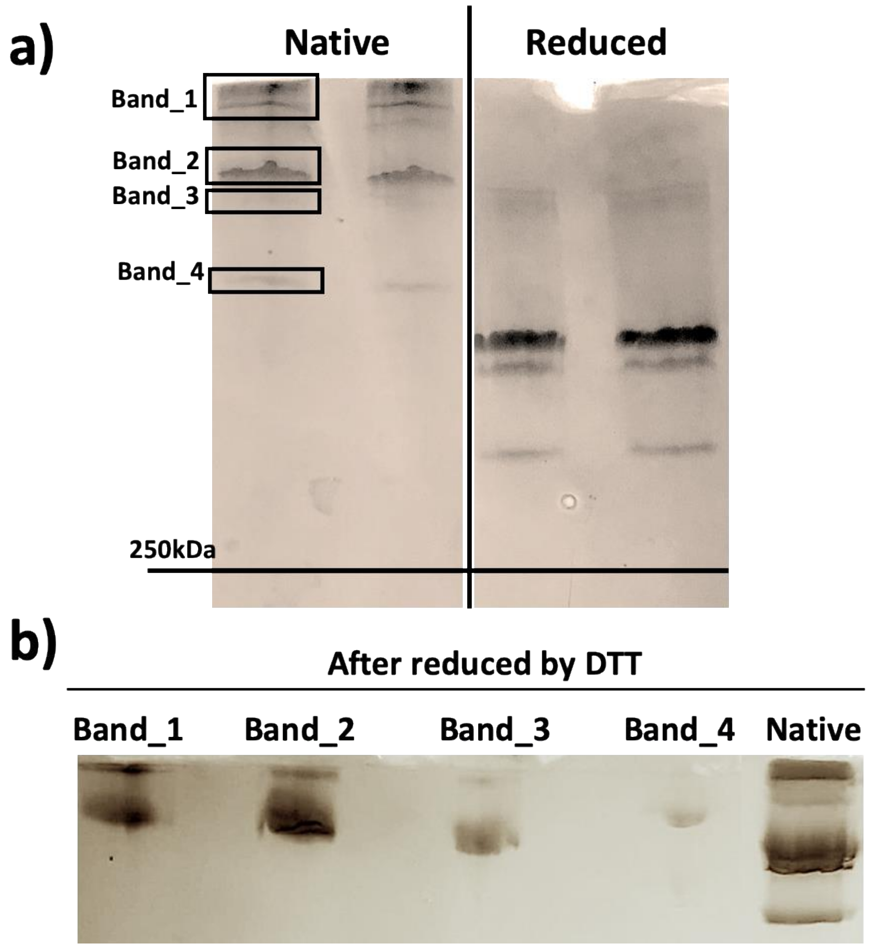

Figure S4. a. Long range SDS-PAGE gel of native and reduced slime by DTT in duplicate. **b.** Second SDS-PAGE gel on the 4 bands cut off from the native sample in the long range gel, subjected to disulphide bond reduction. All bands from the second SDS-PAGE gel were cut off for LC MS/MS analysis.

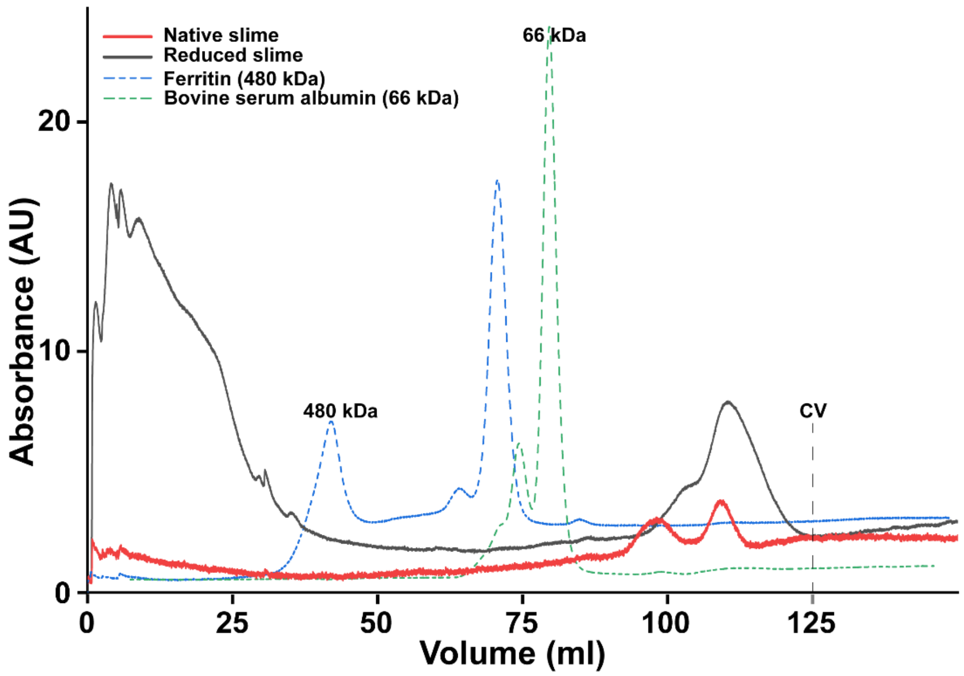

Figure S5. Size-exclusion chromatogram for native slime (red) and reduced slime (grey). Ferritin (blue) and bovine serum albumin (green) are used as molecular weight references. 125 ml equals to one column volume.

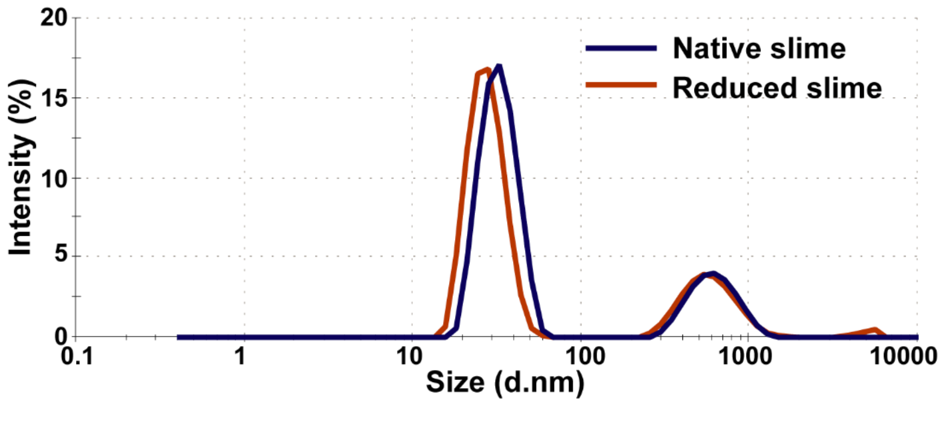

Figure S6. Dynamic light scattering (DLS) spectra of redissolved slime in native and reduced conditions. The size of the redissolved nanoglobules remains similar after reduction.

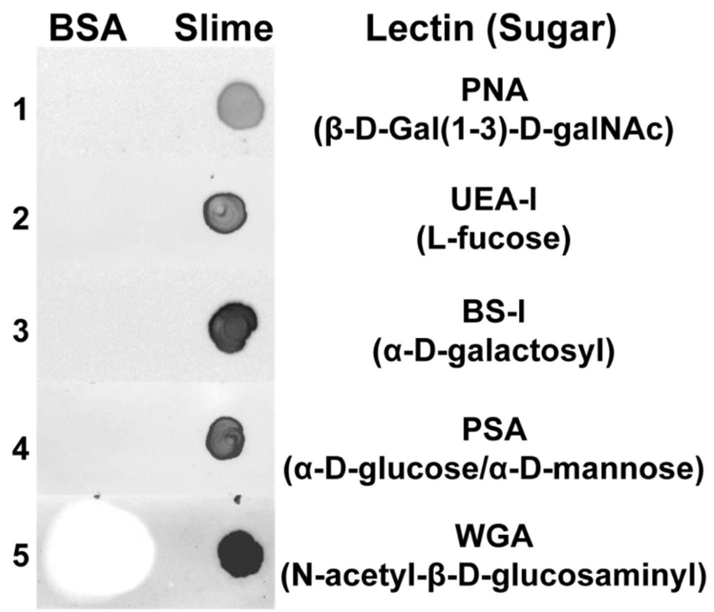

Figure S7. Dot plot assay for detection of lectins binding to slime. Peanut agglutinin PNA, *Ulex europaeus* agglutinin UEA-I, *Bandeiraea simplicifolia* lectin-IBS-I, *Pisum sativum* lectin PSA, and wheat germ agglutinin WGAwere applied to ES slime.

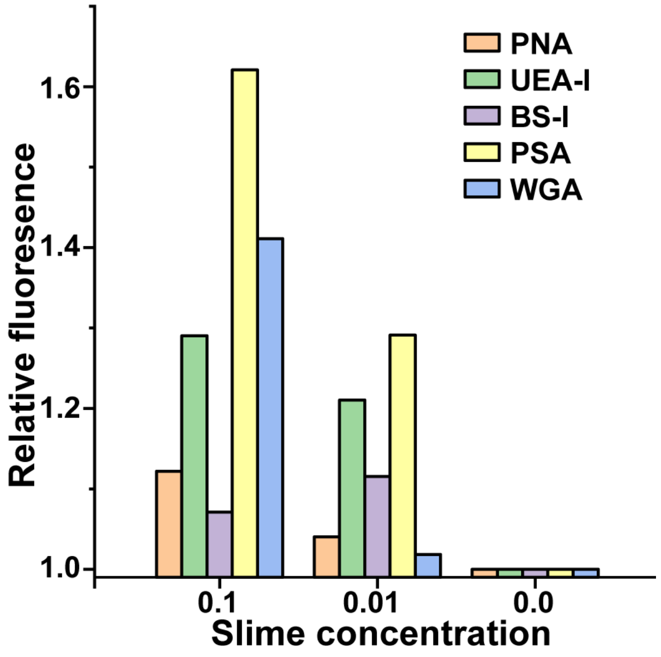

Figure S8. Fluorescence measurements for detection of lectins binding to slime. Peanut agglutinin PNA, *Ulex europaeus* agglutinin UEA-I, *Bandeiraea simplicifolia* lectin-IBS-I, *Pisum sativum* lectin PSA, and wheat germ agglutinin WGA were applied in the lectin assay to ES slime. PSA and WGA bound more strongly to increasing concentrations of slime.

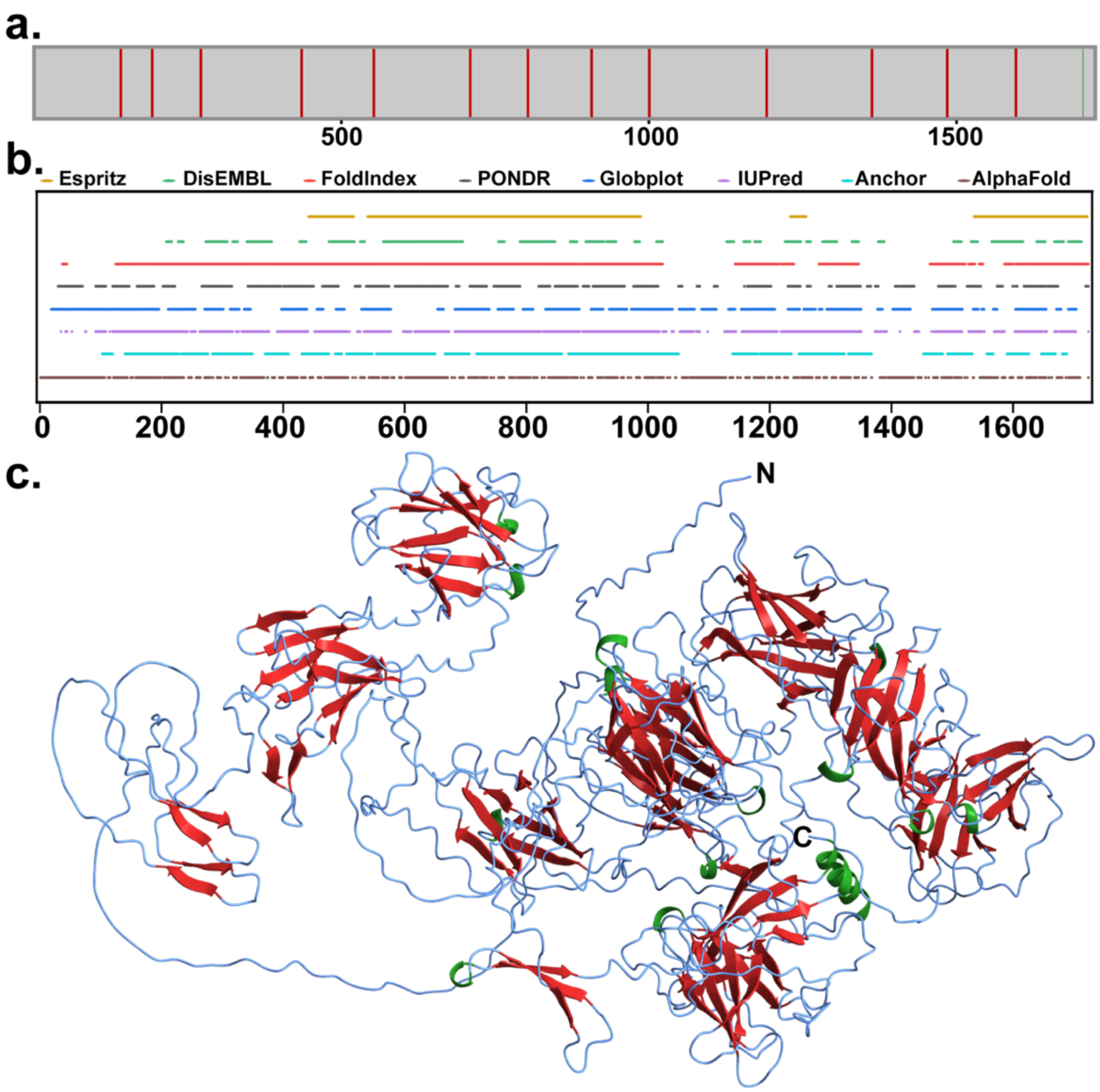

Figure S9. Structural predictions of ES_P2. **a**. Prediction of secondary structure domains predicted by AlphaFold indicated as straight line along the sequence top, green: α-helix, red: β-sheet b. intrinsically disordered regions within ES_P1 using bioinformatics tools and c. Predicted structure of ES_P1 based on AlphaFold, with regions of secondary conformation mapped within the structure green: α-helix, red: β-sheet.

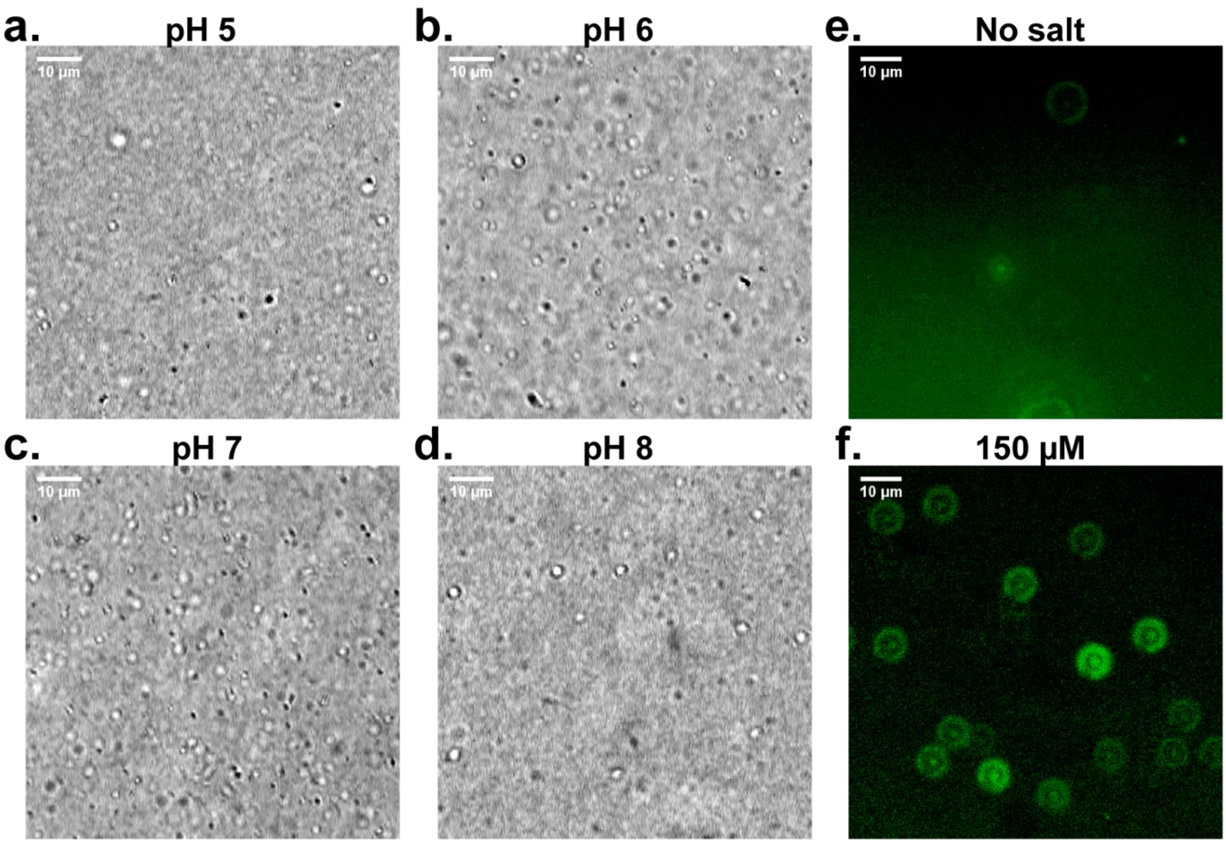

Figure S10. a-d. Microdroplets of 60 µM $ES\_P1_{31\text{-}83}$ recombinant protein observed by optical microscopy at pH 5 (a) , 6 (b) , 7 (c), and 8 (d). **e-f.** GFP encapsulated within $ES\_P1_{31\text{-}83}$ droplets observed under fluorescence microscopy in the absence (e) and presence (f) of salt at 150 µM concentration.

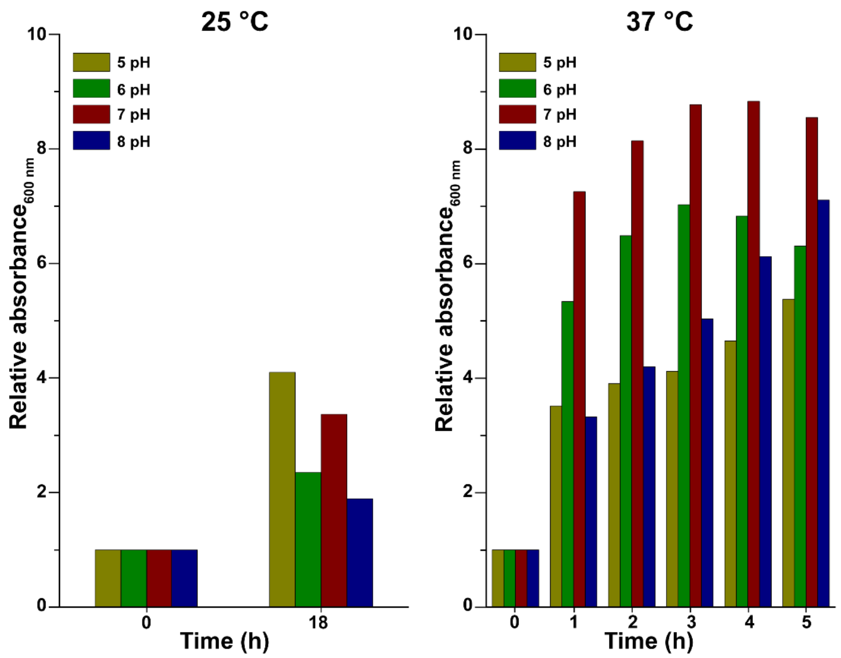

Figure S11. Relative increase in turbidity (absorbance at 600 nm) for ES_P1$_{31\text{-}83}$ at room temperature (25 °C) and 37 °C in the pH range 5 to 8 (citrate-phosphate buffer).

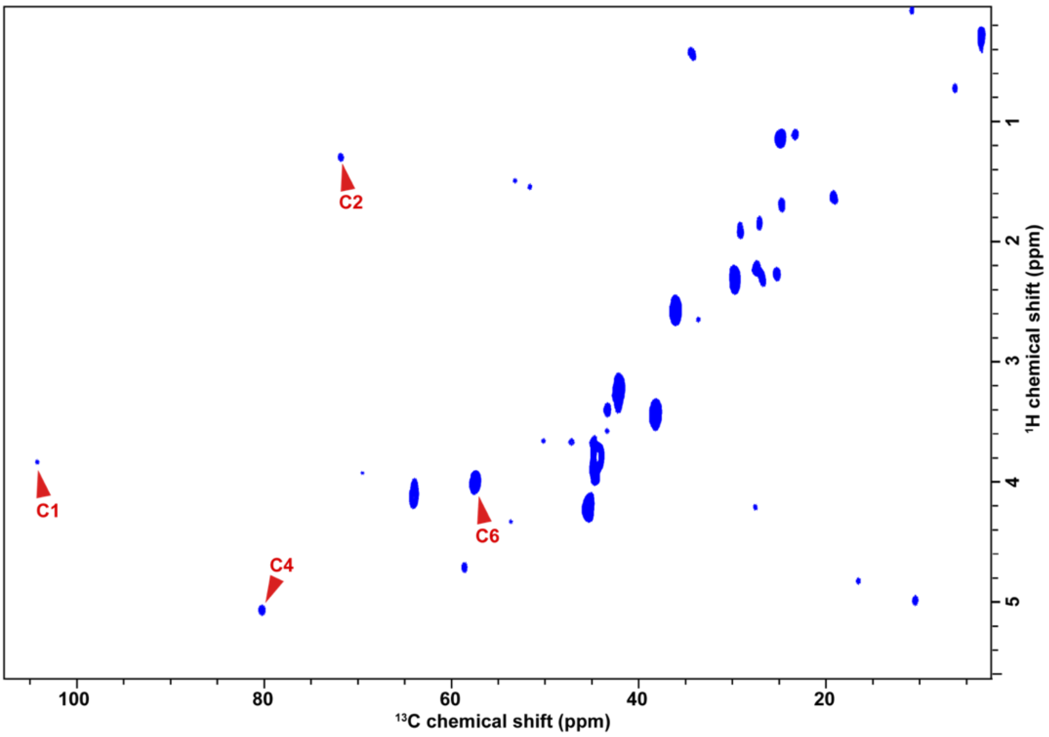

Figure S12. Zoomed-out of the 2D INEPT spectrum indicating carbohydrate peaks with ^{1}H and ^{13}C chemical shifts within 60-110 ppm.

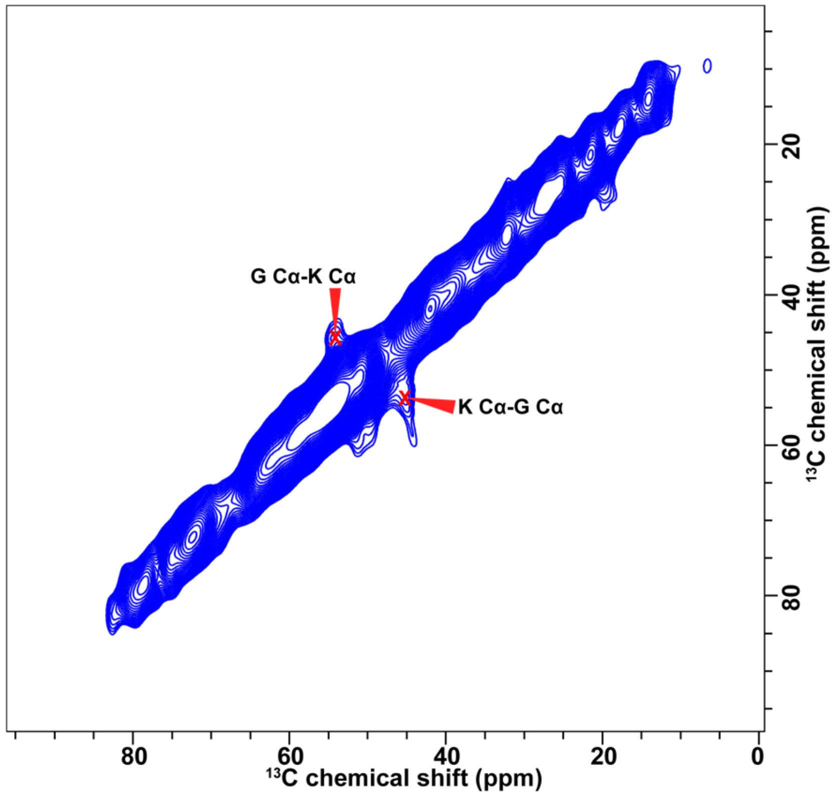

Figure S13. 2D ^{13}C-^{13}C DARR spectrum with 500 ms mixing time showing G/K correlation cross-peaks along the diagonal.

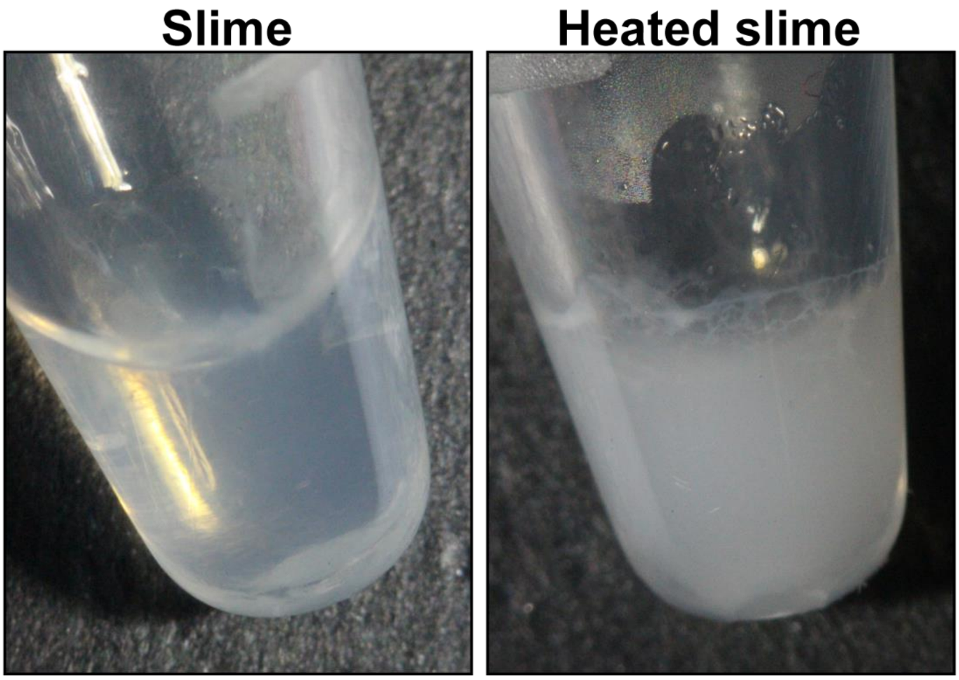

Figure S14. The appearance of re-dissolved slime before and after heating at 70 °C for 10 min.

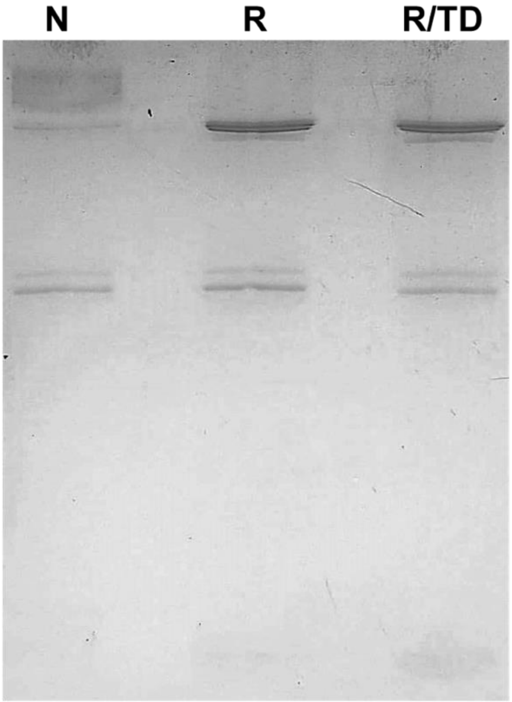

Figure S15. SDS-PAGE gels showing complete reduction of disulphide bond at high DTT concentration. The gel lanes indicate native slime (N), slime reduced with DTT (R), and reduced slime followed by thermal denaturation (R/TD).

Supplementary Movies

Movie S1. Fiber drawn from native slime.

Movie S2. Heat inhibits fiber formation.

Movie S3. Reduced disulphide bond does not inhibit fiber formation.

Movie S4. Combination of heat and reduced disulphide bond inhibit fiber formation.

Supplementary Data

Protein Sequences:

>ES_P1 (hydroxyprolines are highlighted in grey).
MKILLSVLVLLIVVECGNSRKIRHRGGSRRGSGGGSGGSSGGSSGGSDGSYGGSDGGSGGSYGDSGGGS
GDSTGSNGGPGDSYSESGGSSGDGGSGGSYGGSDGGPGGSYGGSGGSGGGGGGGGGGGGGGSGSD
NNPPEGYYDPNKSGPLPPGFEAPPGYEGGEWHPGPDGTMVRTIVEEEPGTETTELVPPDYDPQITPVG
QPGSPGYHDIIGVGKPGERGYFKRTPGPSGNPDDYTLEPIKSPENPEPVDPNDPNAPRVIRTKKNKNPTL
QVGNKDNPQYFGLKPDPKNPGHFTLVPRKMHPGKPGHRKPGHPKGGKGKPGRGKPGHGKPGDGGA
LSPEEYEKLKQNPVMKYPVFTLGKPEHRRSFRITQDPNNPNDFSVDPIGNENDPQSPADPNDPEAPEIIP
QGEKGKPDYNVVIGVNPKKKRDYVQMKPDPKNPQQFNFDPIFMEPQQEEPQQEQPTPAGGEPEVFLD
PGSESVTPEEYERLKQNPKIDRKKGSPILHLGYEPYRRPFRIKQNRRNPLDFDVQPIGSDNDPNSKPDKDD
PYKPVVYPLGEKGQPDYSQIIGVGPKDKRNYVRMIPDPKKPGKFEFEPVLLKPGEPIPFVTETEPPPTPTPE
PTVPPPPPTHPPTTKKPEEEEPCFEDLPEDPEFRRVRHPGRRRPYQIISLGPKDHRQHFKKTPKSNNPDDY
DIEPYDPDTPDHKPKADDPYAPEVIPQGQPGTPSFEPILMTGPKDNRKPYKLVHHPENPSRSSFVPVRPL
PKKPGQKHPKFVNAPRQIRPRKKDEPRQPDDEPEEGEPEFTDLPDRPRFKHVKQPKRPYTLISVGKHPHR
RTFKKTPNPNGNPDDYDLEPYDEDTPDHKPNPHDPSVPKVHKNGKKGTPDYQPVIEIGPINKRKLYKIKP
HPSNPNKVEFVPVKKIMRKGKPFFKELPRRTATKRFRPRTTPKPNEDDDVPDDLPYDPEVRPHRTPGQKY
PTQIISVGKPPNRKHFKKIPKPEDPKKFDLEPIDPDDPDEPLDPEDPNTPKVYPNPEGNPVIETGTPDDPK
HHEVNPDPEDPEDPERVSFTPVKRLGEPGEKNPKFEKIPRKSHPFHTKRPEEATAPEPEENEPVFEDIPYN
PTFKTLHRKRPDRPAQIIALGRPPHRQEFIKEPGRSGKPHDFKLSPYDSRRPDKKPDPNNPNKPEVYPARG
KPGKPGYQPPVIKTGPKDDPEYFEIHPDPSDPKNPNKVLFVPVKARPGKPGTRPKFSPKEPRRYHPYHDP
LKPRPTTEEPEDGTEYFVDQPEDPEVTTLFRPLRKYPSQLIAVGKPPNRKYFEKKPGPSGHPKDYTLHPIDP
KHPDKSLNPKDSKTPKCFPGEGKPGTPNYKPPVIQTGSPDNPKFYRVIPNPKDPNNPESVEFIPVKKDKKN
PKKFKPLPRNVRIYHTTKKQATSQPNQTPPHEEGDPYFTDLPDNPVVLTVRKPGSKKPVQILALGKPPHR
KYFEKVPEPGKPDRFTLKPINPNGKPGEPSKVYPGGNKPGSRYPVIEYGKPPKAFEVRPDPLHPNDPTKV
QFVPVKKVPGTDGPSFVPAPRTVGKYHTTRKKHHKTTKEPKEGSVIYDDGPYLPKFYPGKKNTKIVAVGK
PPKRRYFKIIPKPDKKFTLKPIDPQNPDDPNPHPEDTKVYPQPDGHPVFEIPGPNKKPEYFKLVPKKDNPK
EMDFIPVRPVSPGSFTSKPRTTRHYKTTKKSKRTTRPTSRVIFEDIPHNTQVPKKRPNQEHPSTIITVGNEP
NQKHFEVIPDPSGNPKKFTVVPYNPYHPNKPCDPKNPKTPQVFEVPKDYPVIQGPDKKFYRLEPDKHNP
GQYTFVPVTPLDENHKPSNPHKPDVSFVPIPRKWHLRTTPGIDDWLDLSTTPSPKTTTDNLGTGYSVVTK
PEEISEPTTEVDNAETTVGEEELNKEPTEGPMTTEATEQFSTEQPTVPSHTETEVAIELTTAEEIILSTASVT
QPTVAGKTESTPAELTGKSEIPQSTVSTTEAPTVATHTPMSVTTEAIQGTTQGAGVSTESETKGQPTGAT
SGPEEPSKQEPTTSEVSGTGQTASPATSSEAHPRPTAPNVDDVCFKAYDDCKRSRSSYA*

>ES_P2 (**hydroxyprolines are highlighted in grey)**
MEMMYTLFFLLFGIVHGQGDGWVLQPDGSYMSYGDGSSGGSYGSTGGSYDGSGGLYGGSSGGSYGEL
GSGGLFGGSGGGGFGPGGSYGGFDGGLGGSSGGSQGLPGNGWILQPDGSYLKYEGSGGGGGGGG
GGGSGSDGPPGNGWILQPDGSYMKYDTSGEGNQGSAGSYGGNLPPGFVPPPGYSGNEGGWVLQPD
GTYMKTIEQTEPPTEIRYPVNPDYQPDETLESGKKGSPGYSQVVGFGEPGNMNYFKITPGDAENLYDYSV
EPVVSRDNPTHSTDTEHGKVISRGTKGEPDYQPILQVGPRNHRRYYKMLPDSSKPSGFNFIPQRLTGWLK
KQGGDDTSGEVLEPEESEVTEEANEEIPDEPEIEKKNNDFIICIGRPNRRYLRLKRYSNNPHDYSVEPIGSH
DNPDSKPNPNDEEAPEVMTEGTKGNPNYRSIIAVGPKYNRRYVEVLPNSNDPKYSFAPVDVQVQPGE
QATEPNEEDVEYDDLPENPEYKTIPGKKKSYEIFSFGPKNRRVYFRIIRRNPNNPNDIEVFPFKDPEHPDSP

PSSDDPSAPEVIQQGDPNDPEDGPVIAVGPKNKKVYFRLGIRKDKVPKFTPVRRGKRIPGKHRLVFTKKTR
IWHSRPQKKPLVRKPSAPLKFETSAPVEAATDYSPDEVDVNPENPVEKRPYQIITFGPPKKRIYIKKTPGPS
GNPYDYKLEPIGNPEDPNSKPDPKDPDSPEVIETGEPGTPSFAPVVAHGPKQNRKYVIIRPNSENPKDPN
QMEFSPAEPVPGSDPKKPRFRILRAAVPVAPKDQFTDNTPKNPNIKTALKPGDKTRYEVISFGPKNRRIYL
KKTPGPSNKPDDFKLEPIGNPNDLNSKPDPNDPEAPEVIENGQPGTPDYSPVVAFGPKTRRKYIIVKPSPK
NPKEIQFIPAEPEPGSDPKQRRFKPLTKKVLPGLIRPKPPTHPDSPEIKTVEGPHPHQVISFGPENKRIHVKK
SPGPSGDPNDYKLEPLENPFDPNSKPDPQDKDAPAVIETGKPGTPNFLPIVSYGPHDNRKYMSFKPIGNT
IALSPATLVPGSDPKHPAFNIKTTHPVSPQIPSILKDLHIPTHPLIPPIFNQLPPILNPRGINLLPGLLPVISNLA
ELTKPTVQNVEKPNKPSYQVLSFGPLLNRIHIKKTPGPSGKPDDFKLEPIKDPNDPDSKPDPKDPQTPEIIES
GKPGTPDHQQVIAFGPKPQRKYVLVKLNPTDSKKLELSPVEPEPGSDNKQPKFRPVVKPVIPTLPLPSLDL
HNPVINTVEKPNKPHYQVISFGPSTQRIHIKKTPGPSGKPEDYKLEPLGNPDDPDSKPDPKDPNAPEIVDS
GTPGSPDYEPVVAYGPKTARKYVILKPVPLNPKALDLQPAVLEPGSDPKKKKFKVVIQPGISPLLPQILPPF
WPKPSIPYLPKPPPITLPLISHGLQNPIVNNIEEPNKPHMQIISFGPILTRVHIKKILGPTGNPNDYKLEPIGN
PDDPDSKPDSKDLDAPEIIENGKPGTPDYKPVVAYGPKSLRKYVTLQSRPSNPKEIDLEPAELAPGSDAKK
PIFKYLPAIPVFGNKDRNAPEVKTVTDPISKKPVQVISVGPIFKRVHLKKIPGPSGKPNDYKLEPLGNPDDV
NSKPDPKDPDSPEIIENGTPGTPDYAPVVAYGKKIRRKYLKIKSNPLQPNDPTKLLFNRVRLLSPKPGEKTP
TFSESLIPKFNPVCYKQYQDCNDRS*

>ES_P3
MRMKIIILILSLCATTFSSDVTEVADDANPDDLLAQGYVLQPDGSYLKTDETSSTSEQSGTSDQSPDVLKS
QGYVQQPDGTWLKTVEDTETTYDDNEYKNTDYSDGILTVQNDPDVGYFKINKGSSDNENDYSVQAVNS
PSDPSPINNPGKDDPRVYPQGNQGDPDYHNVIGVGKGKDRTFLKMVPEPSRPSGFKFIPQYISGYRPGD
VTNAPEPAPEPAPETSPGSTDDYEEEPNDNPDEPNISDRKGYFIISIGKPNRRFLKLTPSTNNNPHDFSLVP
IKSPSEHNSSPDPNDEEAPEVISNENRGSPDYQPVIAVGPPDNRRFTGVVVNPENPKSFQFVVRDQELQP
GEAQTEPPEDDIEYEDEPPNINYKVITVGGKKNEVISAGPKNQRFHFRITHKSKNQGDIEIKAINSADNIDS
SPDPDDINAPEIVQQGNPEDPNNAPIVGCGPRFRRTYHRLIITKGRARFLPLRRVKIRGRHSFIKRNRRWH
YRRGIKKIARRVIKRRPLLIRRRKVKYNKRVVMHRNKLISHNRGGLLIGHKSGHSLGGKGGRLIGGGRAGN
GMRHSSRVVIRHHSGSKQNGHGLSLGSRSEGHHSLSIGGRGSSNGGGGGSHHGSHHG*

>ES_P4
MLGKTILILLSFCLFIERSYSFMCRCEKLYRRDMVDGKLVVTEINLCPKTVTEKDCPRGLVRNGCGCCPECG
KDLGQSCSNAMLGPGKCGTGLECVGWEEGNMENTIKAGVCQLKK*

>ES_P5
MWNCVRSNNGIMVLLSLLTVKTPITDADSIRALACRALVGLARSEMVRQIISKLPLFTRGELQVLMKEPVL
QDKCLEHVKFCKYASELIEKVTGKPLSSHIESSLAKINKADVVAQTKIVFNEKELLQLIYQHLMNKGYHESAL
SLEKEASLPKNGFTVPGFFGSPPSSSKLARYLFTSVPSASTLSPSIRNHSNTLGGHGSTPIKMNFLTSKNPH
NGLNSNKNVKFKVIRQKSSCGEFQYSPIMKKQNLVKPQLPTVSLDAIITEYLRKQHEHCKNPVVPCPPFSL
FVSHHCPDPMFRNSAPNNITARILRRSAFPKHGGIDGARLNRKHIYSRFRPVRSYRGVEEEGCFCCCAFSH
DDENLYLGTYTGEIKQYNIQTRTEEASYNCHTSALTLLEPSQDGKLMLTSASWGRSLSGLWAFQNNSLEL
MYSFENDHFVEFSKLSQDRIIGTKEETAHIYDVSTGQLVRTLYNADLANNYTKNRATFSPFDDLALSDGVL
WDVQAANPIHKFDKFNPHISGVFHPMGLEIIINSEIWDLRTFHLLHTVPALDQCQIVFNSAGDVMYGAN
HQLDDDNDLSEDSVKSPFGSSFRTFDATDYSNIATIDIKRNIFDLSTDKSDCLLAIIENQGARDGLVEESICR
LYEVGRTKDQDDEQVGGAAGGAGE*

>ES_P6
MGAKYKPDWEDSCTHLICAFANTPKHRQVQKLGGQVVRKEWIVECYKKKRKLPWKMFRLDGDSEEEE
DDEDNDQTYEEGDDEEDEDEEEIRLEKKYSKKHVPKPGPSNLKKTSPKKTTLTFTEKKPSPKKLKLYDEEEN
EENSKKYSSEEDSGDDTEDEIRKVREEENKKPNIQHTKSDNEYEGSTDEDAELEKKKNVKSLSKTIVENGNSS
STKESIFPLPNLPSFFKEKSFFFFGNFDDATRKLLKRYIIAFGGKLENSMDQKVSFVITASDWDEQFEQALN
ENDSLTFVRPQWIFKCKDKGKFVPYQPYVVVPKESSD*

>ES_P7 Phosphorylated sites highlighted in grey
MKTIIWFFISICVADSAIRVSHQYNFKEKPKNQNSRNSIKRTSGGYGAEMDNTGVLINKYPDTPPPFYQM
LPDDFMNPPDYNNNNNNNGGGGINPPTDYSSGSFDPMPPDDFMNPPDYNNNNNNNGGGINPPTDY
SSGSFDPMPPDDFMNPPDYYNNNGDINPSTDYNSDSFDPISVPDYNNNNGDNNNNQENYENNYNQN
LGNNEQIDEEMPPDYVSSDSGYLPPGYIAPPGISRDGWHLLPDGNYVKYIVETEAPIAYEVPISPVDNPEIL
PVGEAGSPGYHDIIGIGEPNIRSYFKRTPGPSGKPDDYTLEPIVSPRKPIPVNPKLRNRPRVIYPEGRNNPPT
LMIGPEGRRKYFKMVPNDDKPGGYDFIPRIKKGSKIPEPSHIITQKPQISETTEETELTTTELLEPPTEEVTES
PDNYDEEPEFDDLPENPQFETHRPGTNHPFTIISLGPKNHRQHFKKIPRSRNPNDYDLEPYDPNTPNHKV
NPRNPYAPKVLNNGIKGTPQFSPVIKTGQPHFPKFYRLVPHPNNPNQMGFVPVTKNILKPGNKKPTFSD
LPRQFKPPRRRRPEEVRDDEPDDGEPEFSDLPEEPHFKTVKQPKRHPYTLISVGKPPHKNTFKKTPGKSG
NPNDYTIEPFDSDTPNNEPDQDNPDPPKVHPHGEKGKPNYSPVIEYGPHKHRKFFRLVPHPTHPNHFGF
IPVKKVNTKKGPHFQDLPRKYQPHRIHSPTTHKPHEGSKVFDDLPDDPSIITRHLPGRRHPTQIISVGKPPH
RRHFEKKPGPSGDPDDYVIEPFDPKNPNKKPNPHNPKSPKVYHGKHPVIKTGSPQKPKYFRVEPDPNHP
HDPNRVKFTPVKPLKKGKNPTFVEIPRRFHIYHTTPKSSEVTTTEPEDDEPVYDYQPERVKIKNIHPPHSKY
PYQIISIGKPPNRQHFLKKPGPSKKPYDYTIEPYDPKTKDHKPHSKNPPKVYPAKGIPGNPDYQPPVIRVGP
KNKPKYYKLKPDPKHPHDPRKIKFIPVIPVPHRPGKFQNLPYHYYHRTTTKEPDESDIIFDDLPENPEVKELK
RPHQKIPSQIISVGKPPYRKHFEKKPNPSGKPDDFEIYPFDPMHPNKKPKPSDDVFPATGKPGTSKYQPPV
IKTGSPQKPKYFRIIPNPDYPNDPKKIIFIPVKPLKRPDSHKLNFIPLKRHYHIYHTTTKKHPKPPRGQTPEPY
DGEPIFVDQPENPKVFQVKDPKHKRPVQIIAVGKKPHRQYFKKIPGPSHKPDDYRILPFDPRKPNGKPDP
KDKSSPKVYPGPPPVIEYGPKHNRKTFKLVPDPKHPNDPTKINFIPVKKISNKPYDNKPHFQNLPRLTRLYH
RTTKHKHPKTTAEPQEGDIVFTDEPYKPKIYTVKEPGRKHPVQIIAVGKPPNRKYFKKVPKPNDPNDFILYP
YDPQNPHENPNPEDDKTPKVYPGDKNPRKPKHRFPVIETGTPQNPNFYEVVPDPKHPGKVNFVPVIPVK
NIEKPEKPLYFIKPTRKFYRYTKSKKTTTPEPKEGEPQFIDVPDTKVEQKVVPGYKKPVQIVEVGERPYTKKF
VKIPGPSKHPDDYILKPFDRKHPEKNPKTPKVYGPFGKPNTPDYHHPVIEFQPNKFFEVKPNPKYPHDPKK
IILVPVKPTKPKDKNPSFVELPRKWYCYHTTFEPEKEIIEGTTNPEKLTTIQEATTEELITVNDGTIVTITEAEL
TEELTDNPEKKESEVPEKTEEPEFNSTPKPNQSEEVEEPEEPGYPKPQKPSGTGEAEEPERSEESEEPGQKP
RHPKPHRPGSGGEPEMPGAENEGPEEPEKPREPGTGEQEKPGEPETEEPEYKPRHPKSQRPGEENEEPK
EPEKPREPGTGEPEGHEELEKPGEPERPGNKPRHPKPQRPGGENEEPEEPEKPREPEGSGHKPQRPGGE
PGEPEEPGQPEEPGHKPRHPKPQK*
